# Contemporary Challenges of Regenerative Therapy in Patients with Ischemic and Non-Ischemic Heart Failure

**DOI:** 10.3390/jcdd9120429

**Published:** 2022-12-01

**Authors:** Marko Banovic, Gregor Poglajen, Bojan Vrtovec, Arsen Ristic

**Affiliations:** 1 Cardiology Department, University Clinical Center of Serbia, 11000 Beograd, Serbia; 2 Belgrade Medical School, 11000 Belgrade, Serbia; 3 Advanced Heart Failure and Transplantation Center, Department of Cardiology, University Medical Center Ljubljana, 1000 Ljubljana, Slovenia; 4 Department of Internal Medicine, Medical Faculty Ljubljana, University of Ljubljana, 1000 Ljubljana, Slovenia

**Keywords:** stem cells, heart failure, therapy

## Abstract

It has now been almost 20 years since first clinical trials of stem cell therapy for heart repair were initiated. While initial preclinical data were promising and suggested that stem cells may be able to directly restore a diseased myocardium, this was never unequivocally confirmed in the clinical setting. Clinical trials of cell therapy did show the process to be feasible and safe. However, the clinical benefits of this treatment modality in patients with ischemic and non-ischemic heart failure have not been consistently confirmed. What is more, in the rapidly developing field of stem cell therapy in patients with heart failure, relevant questions regarding clinical trials’ protocol streamlining, optimal patient selection, stem cell type and dose, and the mode of cell delivery remain largely unanswered. Recently, novel approaches to myocardial regeneration, including the use of pluripotent and allogeneic stem cells and cell-free therapeutic approaches, have been proposed. Thus, in this review, we aim to outline current knowledge and highlight contemporary challenges and dilemmas in clinical aspects of stem cell and regenerative therapy in patients with chronic ischemic and non-ischemic heart failure.

## 1. Introduction

Even in the 21st century, heart disease represents the leading cause of death worldwide regardless of gender, race or ethnicity [1]. The myocardium has little capacity to regenerate which makes it very sensitive to injury, as seen in acute myocardial infarction (AMI). Any acute myocardial injury typically leads to the loss of cardiomyocytes and replacement fibrosis, ultimately leading to the development of chronic heart failure (HF). Even today, the prognosis for the majority of patients with chronic HF is poor, with 50% of patients having a survival rate of only 3–5 years [2,3]. Indeed, the World Health Organization recognizes HF as a leading cause of morbidity and mortality and one of the most prominent challenges to public health in the coming years [4]. For chronic HF patients, recurrent hospitalizations and high mortality rate represent major unmet issues that necessitate novel treatment options in order to improve the outcomes of this patient cohort. Measures such as left ventricular assist devices or heart transplantation are often the only options that improve the outcomes of patients with advanced HF. However, a very limited number of patients can benefit from these complex and costly procedures, as the number of candidates far exceeds the number of those who can actually access these therapies [5]. Hence, this unmet need opened a space for novel concepts and strategies for the treatment of symptomatic/advanced HF patients [6]. The recognition that the heart is not a terminally differentiated organ created a basis for a new therapeutic option of myocardial regeneration that aims to achieve the structural and functional restoration of a failing myocardium.

It has now been almost 20 years since first clinical trials of stem cell therapy for heart repair were initiated. Initial excitement created by promising preclinical data resulted in the rapid translation of stem cell therapy into clinical trials, which have covered the use of both first (skeletal myoblasts, bone marrow mononuclear cells, hematopoietic stem cells, endothelial progenitor cells, and mesenchymal stem cells) and second generation stem cells (lineage-guided cardiopoietic cells, cardiac stem/progenitor cells, and pluripotent stem cells), as well as a combination of different stem cell types [7]. However, the research road so far has been very turbulent, filled with hopes, disappointments and controversies. While available data suggest that stem cell therapy is feasible and mostly safe, the results of clinical trials have demonstrated inconsistent patient benefits from stem cell therapy regardless of the cell type used. Moreover, the cardioprotective mechanisms of stem cell therapy have become a focus of intense research in recent years. The initially proposed mechanism of de novo myocardiogenesis, based on the pre-clinical data, was never confirmed in the clinical setting. Currently, stem cell paracrine effects on the target myocardium and improvements in microvascular dysfunction are believed to represent the main modes of action of stem cell therapy [8,9,10,11]. Importantly, in the rapidly developing field of stem cell therapy for chronic HF patients, relevant questions regarding protocol streamlining, optimal patient selection, stem cell type and dose, and the mode of cell delivery also remain largely unanswered.

This review aims to summarize current knowledge and highlight contemporary challenges and dilemmas in clinical aspects of stem cell and regenerative therapy in patients with chronic ischemic and non-ischemic HF.

## 2. Pathophysiologic Changes in Ischemic Heart Failure

The modern management of AMI with rapid or early revascularization has significantly reduced intrahospital and early cardiovascular mortality but has precipitated an increase in the incidence of HF in survivors [12]. Acute myocardial ischemia leads to the rapid and irreversible loss of cardiomyocytes. It is estimated that a loss of 25% of the myocardium corresponds to a loss of roughly one billion cardiomyocytes [5]. Because of the limited regenerative capacity of the myocardium, the remaining cardiomyocytes are unable to sufficiently proliferate and replace a lost myocardium. In comparison to primary percutaneous coronary intervention in the AMI setting, such a rapid life-saving therapy has not been developed for chronic ischemic cardiomyopathy. During the late post-AMI phase (>72 h), activated fibroblasts begin the process of adverse myocardial remodeling in which systemic (renin–angiotensin–aldosterone system, and sympathetic activation) and myocardium-specific (inflammatory cells, proinflammatory cytokines, growth and paracrine factors, matrix metalloproteinases, tissue inhibitors of metalloproteinases, etc.) mechanisms are also crucially involved [13,14]. The fibrotic replacement of an ischemic myocardium, the cellular and macroscopic remodeling of the remaining (non-ischemic) myocardium (that acquires a spherical shape), a decrease in capillary microcirculation density, and impaired excitation–contraction coupling all further compromise the structure and function of a viable myocardium and underscore key features of chronic ischemic HF [15]. Left untreated, this vicious cycle ultimately leads to advanced HF and death. Yet, major improvements have been made in last few years with regard to the drug and device treatment of advanced HF, targeting systemic and myocardium-specific mechanisms involved in HF. Although new drugs, as well as implanted devices (such as cardiac resynchronization therapy or left ventricular assist devices) prod reverse remodeling [5], these therapies not only differ from each other in the mechanism of action but are also, in some ways, distinct from the regenerative mechanism of stem cells. Overall, new drugs such as angiotensin receptor neprilysin inhibitors and SGLT 2 inhibitors cause diuresis, natriuresis, vasodilatation, the inhibition of the renin–angiotensin and sympathetic nervous system, and metabolic changes. However, contrary to stem cells, they do not directly target a necrotic and scarred myocardium and thus do not lead to its complete recovery. Instead, they target the viable part of the myocardium in an effort to preserve/enhance its contractility and promote reverse myocardial remodeling [2,6,8]. This is important, as it opens up the possibility of stem cell application on top of standard-of-care for HF medical management, with repopulating a necrotic/scarred myocardium with new myocardial tissue being the main goal of this novel treatment approach. 

## 3. Mechanism of Stem Cell Action in Ischemic HF and Cardiomyocyte Regeneration

It is now established that the heart is not a terminally differentiated (postmitotic) organ. Bergmann et al. carbon-14-dated myocardial cells, including cardiomyocytes, and determined that cardiomyocytes renew at rate of ≈1% per year at the age of 20, with the regeneration rate gradually decreasing with patient age, so at an age of 75 years, approximately 0.3% of the cardiomyocytes are renewed per year [16]. All in all, less than 50% of the myocardium is regenerated during an average lifetime. While endogenous myocardial regeneration is insufficient to compensate for a significant myocardial injury, it represents a basis of the stem cell therapy for a failing myocardium. 

Currently, the exact mechanisms of stem cell-associated myocardial regeneration are still not fully known, and several potential modes of stem cell action have been proposed over the past two decades. As injected adult stem cells (regardless of the cell type) likely do not engraft into a failing myocardium in a sufficient quantity to generate a meaningful quantity of de novo cardiomyocytes and thus generate new myocardial tissue, neo-myocardiogenesis (though shown in the preclinical setting) was dismissed as a relevant mechanism of stem cell therapy in HF patients [17,18]. Another theory suggested that stem cells can stimulate resident cardiac stem cells to generate new cardiomyocytes. This theory has been put under scrutiny because several lineage-tracking studies using complimentary techniques could not demonstrate that cardiac c-Kit+ stem cells indeed generated new cardiomyocytes [9,11]. In vitro studies implied that another stem cell type, Sca-1+, may differentiate into cardiomyocytes [19,20]. However, subsequent studies failed to confirm these initial observations, because the transplantation of Sca-1+ cells into an infracted myocardium did not induce their differentiation into cardiomyocytes and genetic lineage tracing based on Sca-1 knock-in Cre lines mainly revealed the differentiation of injected stem cells into endothelial (vascular) and not myogenic components of the myocardium [21,22]. These data suggest that stem cells mainly influence myocardial regeneration through a paracrine-mediated mechanism, as stem cells have been shown to secrete multiple protein factors, exosomes, and microRNAs [23]. This hypothesis is supported by data showing that mesenchymal stem cell (MSC) therapy is associated with the inhibition of the synthesis of proinflammatory cytokines, such as TNFα and IL-1, which have a detrimental effect on cardiomyocytes and induce apoptosis [24]. MSCs are further associated with increased tissue levels of the anti-fibrotic cytokines such as hepatocyte growth factor (HGF) and prostaglandin E2 (PGE2) [24]. Additionally, MSCs can be re-programed to release specific stimulatory or inhibitory molecules by means of preconditioning and genetic manipulation [25]. Recently, the latter hypothesis was challenged by the data of Vagnozzi et al. [26], who, in a preclinical model of ischemia–reperfusion myocardial injury, compared the acute immune response to two distinct types of adult stem cells (bone marrow mononuclear cells and cardiac progenitor cells), cells killed by freezing and thawing, and a chemical inducer of the innate immune response (zymosan) [26]. Their data suggest that cell therapy mainly exerts its salutary effects on a failing myocardium by stimulating the responses of specific types of macrophages that, in turn, differentially affect the passive mechanical properties of the injured myocardium [26]. Importantly, the authors found no difference in the type and the magnitude of the immune response between the animals receiving live stem cells and the fragments of dead stem cells [26]. While the leading pathophysiological mechanism of stem cell action remains unresolved, it is likely that several complementary mechanisms may contribute to the salutary clinical effects of cell therapy in different heart failure patient cohorts (acute vs. chronic; ischemic vs. non-ischemic HF).

The novel and promising stem cell types, explored for use in myocardial regeneration, include use of allogeneic stem cells and pluripotent stem cells (embryonic stem cells and induced pluripotent stem cells), as well as different approaches for a new generation of regeneration therapy. The use of allogeneic stem cells (i.e., from healthy donors) could hold more promise than autologous application (discussed in more detail later in this manuscript) [17]. Initial clinical data suggest that the use of allogeneic stem cells in chronic HF patients is feasible, safe and potentially effective [27]. 

In comparison with adult stem cells, pluripotent stem cells can indefinitely divide and can differentiate into any cell type, including cardiomyocytes. These characteristics are also aggravating for the therapeutic use of pluripotent cells because their growth and differentiation are difficult to control, possibly resulting in teratoma formation and malignant ventricular arrhythmias [28,29]. Contrary to natural embryonic stem cells, induced pluripotent stem cells (iPSCs), pioneered by Yamanaka et al. [30], have been reprogrammed from somatic cells (i.e., fibroblasts) to become pluripotent. However, the insufficient maturation of human iPSC cardiomyocytes (they exhibit fetal-like phenotype) and the risks of malignancy and arrhythmogenesis currently still preclude the wider use of these cells in stem cell clinical trials.

## 4. Clinical Effects of Stem Cells in Ischemic HF

Several clinical trials have been performed in the setting of ischemic HF since the data from the first clinical study were published in 2003 [31]. A comparison of these trials is difficult because the cell types, application methods, number of injected cells, patient clinical characteristics, study designs, and endpoints have widely differed from study to study. In most studies, adult stem cells were used (predominantly bone marrow-derived mononuclear cells but also skeletal myoblasts, adipose-derived stem cells, blood-derived endothelial progenitor cells, cardiac stromal cells, etc.) [32].

Among the most often used bone marrow-derived cells are hematopoietic cells (HCs), mesenchymal stem cells (MSCs), and endothelial progenitor cells (EPCs), of which MSCs have shown the most promising therapeutic potential [11]. Due to their significant arrhythmogenic potential, skeletal myoblasts, initially considered to have significant potential in myocardial regeneration, are no longer in use. Despite the lack of long-term data, MSC-based stem cell therapy in ischemic HF patients has demonstrated better clinical effects in comparison with other studied stem cell types [25,33]. In the MSC-HF (Autologous Mesenchymal Stromal Cell Therapy in Heart Failure) trial, the intramyocardial application of MSCs significantly improved left ventricular end-systolic volume, ejection fraction, and myocardial mass in patients with ischemic HF [34]. The recently published HUC-HEART phase II trial showed that the intramyocardial delivery of umbilical cord-derived MSCs was advantageous in comparison with the transplantation of bone marrow-derived mononuclear cells or CABG alone in patients with ischemic HF [35]. In the POSEIDON trial [36], treatment with autologous MSCs was not associated with improvements in LVEF, 6-minute walk test, or Minnesota Living with HF Questionnaire, but the treatment with allogeneic MSCs did demonstrate significant improvements in myocardial function, exercise capacity, and patients’ quality of life.

In terms of the clinical outcomes of autologous stem cell therapy in patients with ischemic HF, the data in the literature are inconsistent. Evidence from different randomized phase II/III trials reporting the effects of cell therapy in patients with ischemic HF [34,35,36,37,38,39,40,41,42,43,44,45] is summarized and shown in Table 1. A recent meta-analysis of 669 patients showed evidence that stem cell therapy may be efficacious in ischemic HF patients [46]. Currently, the most significant factor limiting the efficacy of stem cell therapy in patients with ischemic HF is the inability of stem cells to replace scar tissue in a failing myocardium with functional cardiomyocytes [47]. Although some clinical studies [37,38] have shown improvements in left ventricle systolic function after stem cell therapy, this has largely been achieved at the expense of the enhanced contractility of a non-damaged myocardium rather than through the remuscularization of scar tissue and the replacement of lost cardiomyocytes in the infarcted area.

In the future, this limitation of cell therapy might be addressed with the use of pluripotent stem cells. Menasche et al. used human embryonic stem cell-derived cardiac progenitors (ESCORT trial, NCT02057900) that were surgically implanted as epicardial patches in chronic ischemic HF patients [48]. During the median follow-up of 18 months, no cardiac or extra-cardiac tumor formation or arrhythmias were observed, providing a proof-of-concept that a fibrotic and scarred myocardium could be replaced with new cardiomyocytes and result in the improved global and segmental function of a failing myocardium.

## 5. Preclinical Studies Using Pluripotent Stem Cells for Cardiomyocyte Regeneration

Although there have been no direct comparisons (except for a few experimental studies [49,50]), second-generation lineage-guided stem cells (such as cardiopoietic cells) and pluripotent stem cells may be superior to adult stem cells in terms of efficacy [51]. Recent data suggest that [37,39,52] cardiopoietic cell-mediated proteome alteration could augment smooth muscle and endothelial cell protein expression, induce the development of vascular components (endothelial and smooth muscle cells), and restitute the metabolic profile of the myocardium [53]. Furthermore, preclinical data demonstrated that human pluripotent stem cell-derived cardiomyocytes can renew a necrotic myocardium [54]; importantly, the regenerated myocardium was preserved for the entire duration of the study (3 months). A histological examination confirmed that the tissue was vascularized and electrically coupled to the surrounding myocardium. Although a significant amount of ventricular arrhythmias was observed and the follow-up was not long enough to exclude the possibility of teratoma formation, the results of this study support the initial goal of stem cell therapy—the restoration of a lost myocardium and its functional integration into the preserved surrounding myocardium.

Human induced pluripotent stem cell (hiPSC)-derived cardiomyocytes have also been studied in preclinical settings and yielded promising results. In a porcine model, the injection of human iPSC-derived cardiomyocytes, together with human iPSC-derived vascular cells, has been successful in reducing cardiomyocyte necrosis and increasing endogenous cell survival [55]. In a preclinical study by Liu et al., the direct surgical injection of 750 million cryopreserved human embryonic stem cell-derived cardiomyocytes (hESC-CMs) improved systolic function and remuscularized the replacement fibrosis in a failing myocardium [56]. One month after hESC-CM implantation, the left ventricular ejection fraction was significantly improved by 10.6 ± 0.9% in the study group vs. 2.5 ± 0.8% in controls. Furthermore, after at 3 months, there was an additional +12.4% improvement in the left ventricular ejection fraction in the treatment group vs. a −3.5% decline in controls. Challenges associated with use of hiIPCs include insufficient cardiomyocyte differentiation (they typically exhibit a fetal-like phenotype, [57] and risks of tumorigenesis and arrhythmogenesis after implantation [58]. Numerous strategies have been developed to ameliorate the maturity of hiPSC-derived cardiomyocytes and include increasing culture stiffness (on polyacrylamide hydrogel substrate [59]), using different biochemical and electrical stimulations [60,61], and developing platforms with biomimetic 3D microenvironments [62]. Given that it takes time to generate a sufficient quantity of hiPSC-derived cardiomyocytes (approximately 6 months) for a single treatment, it is most likely that these cells might only be applicable in the setting of chronic HF [17]. The use of pluripotent stem cells may represent a promising approach to myocardial regeneration, but more preclinical data are needed to address the safety of (induced) pluripotent stem cells before introducing this stem cell type into clinical trials.

## 6. Pathophysiologic Mechanisms of DCMP

Non-ischemic dilated cardiomyopathy (DCMP) is defined by characteristic morphological and functional changes of a failing myocardium, the hallmark being left ventricular (or biventricular) dilation accompanied by ventricular systolic dysfunction. This occurs in the absence of extrinsic factors that may increase the ventricular preload and/or afterload (coronary artery disease, arterial hypertension, valvular heart disease, or congenital heart disease) that can, in turn, result in a similar HF phenotype. DCMP is found in all age groups, has a world-wide distribution (is found in all races), and occurs in both genders [63]. The etiology of DCMP is currently incompletely understood but is likely to be multifactorial, being influenced by genetic factors, infectious diseases (commonly viral), autoimmune and toxicity-related causes, and excessive mechanical stress [63]. Gross organ inspection may reveal the enlargement of all four cardiac chambers with a relatively more pronounced dilation of left or both ventricles. A histological analysis of a failing ventricular myocardium may demonstrate areas of cardiomyocyte necrosis and/or apoptosis that may be accompanied by signs of tissue inflammation and fibrosis. Of interest, the quantity and the distribution of myocardial fibrosis in DCMP patients appears to be different than in patients with ischemic heart disease, being less in quantity and more diffuse in distribution (perivascular and interstitial pattern) in the former group [64].

Importantly, recent data suggest that defective vascularization, impaired vasculogenesis, and angiogenesis can also occur in patients with DCMP [65], underscoring the idea that, in addition to myocardial inflammation, microvascular ischemia may represent one of the key pathophysiological mechanisms involved in the occurrence of DCMP and its progression to end-stage HF. While the mechanisms that lead to alterations of vasculogenesis and angiogenesis in DCMP remain largely unexplained, it appears that impaired myocardial homing (principally managed by SDF-1/CXCR4 axis), the survival of circulating CD34^+^ and endothelial progenitor cells (EPC) in the earlier phases (NYHA class I and II) of disease, and the exhaustion of the pools of these progenitors in the later phases (NYHA III and IV) of the disease may be critically involved [66,67,68].

## 7. Cell Types and Their Clinical Effects in DCMP Patients

In the last decade and a half, several stem cells types have been studied for the treatment of patients with chronic HF [69]. For the most part, these studies (Table 2) have considered the clinical effects of cell therapy in patients with chronic ischemic HF, and only a handful of clinical trials have specifically addressed the clinical effects of cell therapy in the DCMP patient cohort.

Hematopoietic and non-hematopoietic stem cells found in bone marrow (BMMCs) have both been shown to have the potential for trans-differentiation into different cell lineages conditional to a tissue-specific cytokine environment. With accessibility and straightforward procurement, BMMCs have understandably gained the widest attention in preclinical and early clinical trials of cell therapy in HF. BMMCs have, for the most part, been analyzed in the setting of ischemic HF. Nevertheless, several studies have also addressed the safety and efficacy of these cells in the DCMP patient cohort. 

In a pilot trial to assess the potential effects of selective intracoronary bone marrow-derived progenitor cell infusion in patients with nonischemic dilated cardiomyopathy (TOPCARE-DCM study), the intracoronary infusion of a BMMC cell suspension was performed in 33 DCMP patients using an over-the-wire balloon catheter. In this trial, BMMC cell therapy was associated with an improved regional wall motion of the injected myocardial area and an increase in global left ventricular myocardial performance (increase in LVEF by 3%). What is more, persistently decreased NT-proBNP levels at a 12-month follow up suggested a likely beneficial effect of BMMC cell therapy on the LV remodeling process [70]. In a percutaneous intracoronary cellular cardiomyoplasty for nonischemic cardiomyopathy study (ABCD study), 44 DCMP patients received either the intracoronary infusion of a BMMC cell suspension (24 patients) or a sham infusion (20 patients) [71]. While LVEF improved in the treatment arm by 5.4% at a 3-month follow-up, it remained unchanged in the control arm. Additionally, an improvement in the NYHA functional class was seen in the treatment arm but not in controls [71]. These results were further supported by a study of refractory DCMP patients in which the intracoronary infusion of a BMMC cell suspension was also associated with an improved performance of a failing myocardium, increased maximal oxygen consumption, and better quality of life [72]. Finally, a randomized trial of combination cytokine and adult autologous bone marrow progenitor cell administration in patients with non-ischemic dilated cardiomyopathy (REGENERATE-DCM study) corroborated these encouraging early results by demonstrating a 5.4% increase in the LVEF of 15 DCMP patients who received the intracoronary infusion of a BMMC cell suspension. This improvement in the performance of a failing myocardium was further associated with a decrease in the NYHA functional class, a reduction in neurohumoral activation, and improved patients’ exercise capacity. What is more, all of the mentioned benefits, associated with cell therapy, persisted for the entire duration of the 1-year follow-up period. Of note, REGENERATE-DCM also explored the putative actions of G-CSF stimulation on LVEF and found no correlation, resolving the argument that peripheral G-CSF stimulation is itself sufficient to promote the homing and engraftment of circulating stem cells to a failing myocardium [73].

Taken together, the available data support the potential clinical benefit of BMMC cell therapy in DCMP patient populations. As is the case in patients with ischemic cardiomyopathy, the differences in patient selection criteria and cell delivery methods used in DCMP studies make any direct comparisons between cell efficacy difficult and any extrapolation to a wider clinical utility very challenging.

Hematopoietic stem cells (HSCs) (part of the hematopoietic cell compartment of the bone marrow) differentiate along lymphoid and/or myeloid lineages to form mature leukocytes. HSCs are positive for the CD34^+^ surface marker and have the potential to differentiate into endothelial cells and therefore to promote the neovascularization of target tissues [79]. The capacity of CD34^+^ cells to differentiate into endothelial cells could directly address one of the principal mechanisms involved in the pathophysiology of DCMP. As the procurement of CD34^+^ cells is more complicated and related to more cumbersome logistics and higher costs in comparison with BMMCs, it is of little surprise that this stem cell population has not been extensively explored.

In the first clinical trial to study the clinical effects of CD34^+^ cell therapy in a DCMP population, 28 patients received the intracoronary infusion of a CD34^+^ cell suspension. The latter was procured using G-CSF stimulation followed by peripheral blood cytapheresis and immunomagnetic selection. A 5% increase in LVEF was observed in the CD34^+^ cell-treated group at a 1-year follow-up, whereas no significant changes were observed in controls [74]. Improved patients’ exercise capacity and reduced neurohumoral activation were also observed in the study group compared with controls [74]. Importantly, the positive clinical effects of CD34^+^ cell therapy were shown to persist through the 5-year follow-up period and, even more importantly, to translate into the improved survival of DCMP patients receiving CD34^+^ cell therapy [75]. Our recent data further suggest that transendocardial (TE) cell injections may be preferred to the intracoronary (IC) infusion of cell suspensions in this patient cohort. It was shown that TE cell injections were associated with up to 5-times higher myocardial cell retention rates (ca. 18%) compared with the IC stem infusion of cell suspensions (around 4%). This increase in the myocardial cell retention rate translated into the significantly better functional recovery of a failing left ventricle (LVEF improved by 8% in the TE group vs. 4% in the IC group), the better functional recovery of DCMP patients, and a larger decrease in neurohumoral activation in this patient cohort [76]. These results are in line with findings that a percutaneous (transfemoral or transbrachial approach, [80]) intramyocardial injection, or a surgically positioned patch over the epicardium, provides somewhat greater possibilities for cell retention [81]. Whether better myocardial cell retention rates also translate to the increased survival of DCMP patients remains to be defined in future trials. The recently completed repetitive intramyocardial CD34^+^ cell therapy in dilated cardiomyopathy study (REMEDIUM study) evaluated the potential benefits of repetitive transendocardial CD34^+^ cell therapy in DCMP patients. Unfortunately, the study failed to show any conclusive benefits of this approach over single-dose CD34^+^ cell therapy [82].

Cardiosphere-derived cells (CDCs) represent a mix of cells that are extracted from myocardial biopsy tissue specimens and which express both hematopoietic and mesenchymal surface antigens [83]. In vitro CDCs were shown to possess the capacity to trans-differentiate into cardiomyocytes. Preclinical data further suggested that the intracoronary infusion of CDCs may be associated with the functional and structural repair of a failing myocardium [84]. As of July 2022, there are no finalized clinical trials of CDCs in the DCMP patient cohort. 

## 8. Clinical Efficacy of Cell Therapy in DCMP Patients

**Figure 1:**
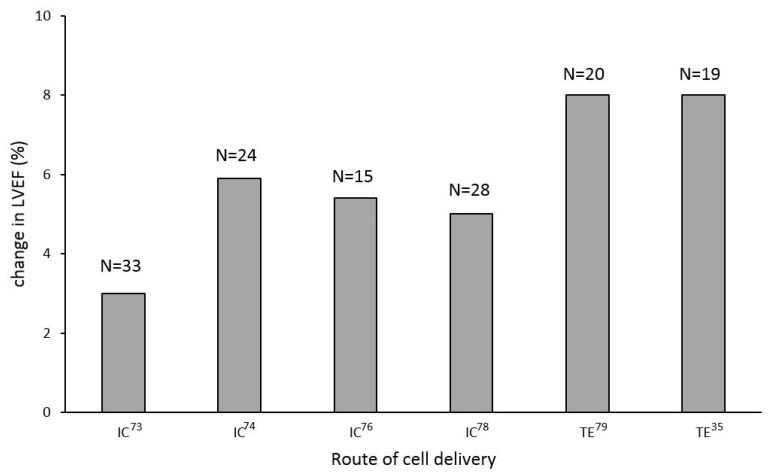
Clinical trials of stem cell therapy in patients with DCMP showed improvements in left ventricular ejection fraction, with transendocardial delivery being seemingly more efficient; TE—transendocardial cell delivery; IC—intracoronary cell delivery.

Studies evaluating cell therapy in the DCMP patient cohort have shown unequivocal signs of improvement in left ventricular function (Figure 1) and exercise capacity [70,71,72,74,78]. Nevertheless, whether these improvements translated into reductions in hospital re-admissions and improved the long-term survival of this patient cohort remain unknown.

The unpublished data from the registry of cell therapy in non-ischemic dilated cardiomyopathy (RECORD registry) on 148 chronic HF patients suggest that cell therapy may be associated with a decrease in hospital re-admissions due to worsening HF, as within 12 months after cell therapy, a significant decrease in HF-related hospital admissions was observed when compared with the 12-month interval before cell therapy (0.8 ± 0.8 admissions/year before cell therapy and 0.5 ± 0.9 admissions/year after cell therapy; *p* = 0.007).

The longest follow-up of DCMP patients receiving cell therapy published to date convincingly showed that at a 5-year follow-up, CD34^+^ cell-treated patients demonstrated significantly lower cardiovascular mortality, being 14% in the stem cell group (55 patients) and 35% in the control group (55 patients). The rate of pump failure-associated death (but not sudden cardiac death) was found to be significantly lower in the treatment arm (5% vs. 18%; *p* = 0.03) [75]. While these preliminary results are based on small amounts of single-center data and certainly need to be confirmed in larger clinical trials, they still offer an encouraging signal that cell therapy on top of optimal medical management may be able to further improve the long-term outcomes of DCMP patients.

## 9. Allogeneic Stem Cells Application in Ischemic and Non-Ischemic HF

Recently, allogeneic cell therapy came to focus in the field of chronic ischemic/nonischemic HF therapeutics to negotiate the limitations of autologous cell therapy [78,85,86]. One of the reasons for this is the wide initiative to standardize the cell products used in stem cell clinical trials. This would enable the production/derivation of commercial cell products that would enable cell therapy in these patients to be employed without the need for bone marrow stimulation and/or aspiration (the latter is invasive) and without stem cell post-processing, which is costly and quite logistically demanding. 

Another clinically more relevant reason is that sicker patients with HF symptoms produce substantially lower numbers of much less potent stem cells [66,67,68]. While the underlying processes of this advanced chronic cardiomyopathy-associated stem cell “suppression” remain ill-defined, systemic inflammation is currently believed to be the main underlying mechanism [72]. Available data also suggest that standard cardiovascular risk factors (male gender and age, arterial hypertension, diabetes, and hyperlipidemia) are further associated with a reduction in circulating endothelial progenitor cells (EPCs) and CD34^+^ cells [87,88]. Additionally, it has been shown that the clustering of these risk factors can further potentiate a reduction in the stem cell count of peripheral venous blood [89]. All of this affects the lower potency of autologous treatment in patients with chronic ischemic/nonischemic HF.

To overcome these limitations of autologous stem cell therapy, trials using allogeneic stem cells were started. In the setting of non-ischemic HF, the randomized comparison of allogeneic versus autologous mesenchymal stem cells for nonischemic dilated cardiomyopathy study (POSEIDON-DCM study [78]) compared the safety and efficacy of the transendocardial injections of autologous and allogeneic MSCs. Compared with autologous MSCs, the authors were able to show significantly better responses to allogeneic MSC products. The latter were associated with better improvements in left ventricular performance (increase in LVEF 8% vs. 4%), decreases in myocardial inflammatory markers, increases in patients’ exercise capacity, and higher quality of life scores [78]. Hare et al. speculated that the greater efficacy of allogeneic MSCs in DCMP patients may be associated with a younger MCS donor age (the mean donor age in the allogeneic MSC group was about 50% of the age in the autologous MSC group) and the possible adverse impact of the systemic proinflammatory milieu of HF on the potency and the viability of autologous MSCs [78]. Furthermore, Butler et al. evaluated patients the safety and efficacy of the intravenous infusion of an allogeneic mesenchymal stem cell suspension (1.5 × 10^6^/kg) in 22 DCMP patients. Although no improvement in left ventricular function was observed at a 90 day-follow-up, the study results suggested better exercise capacities and higher quality of life scores after stem cell therapy [77]. The currently ongoing “the dilated cardiomyopathy intervention with allogeneic myocardial-regenerative cells study” (DYNAMIC study) is aiming to confirm the safety of allogeneic CDCs in DCMP patients, though the results of the trial were expected in 2020.

Additionally promising are preclinical data on the use of allogeneic MSCs in the setting of ischemic HF [90,91]. Accordingly, initial clinical experience was encouraging, as allogeneic stem cells were demonstrated to be safe and feasible in an ischemic HF patient population [89]. Furthermore, in the TRIDENT study [45] conducted in patients with ischemic HF, the transendocardial application of either 20 or 100 million allogeneic MSCs reduced scar tissue, but an LV ejection fraction improvement was only noticed in the group with 100 million cells. In the ALLSTAR trial, the intracoronary infusion of allogeneic cardiosphere-derived cells failed to reduce scar size in patients with subacute/chronic ischemic HF, but the post hoc analysis demonstrated beneficial effects on LV volumes and NT-proBNP serum levels at a 6-month follow-up [29]. Yet, these encouraging data should be interpreted with caution as the trial was terminated prior to completion due to the futility of reaching a primary endpoint and thus did not achieve sufficient power to detect any of the pre-specified secondary endpoints.

In summary, initial clinical experiences suggest that allogeneic stem cell therapy is feasible, efficacious, and safe. This cell type may thus represent an important step towards standardized cell therapy in patients with ischemic and non-ischemic HF. The ongoing phase II multicenter SCIENCE trial (NCT02673164) investigating the NOGA-guided transendocardial implantation of off-the shelf allogeneic adipose-derived stromal cells in patients with ischemic HF [92], as well as a phase III trial evaluating the efficacy and safety of allogeneic mesenchymal precursor cells (Rexlemestrocel-L) for the treatment of patients with both ischemic and non-ischemic cardiomyopathy (DREAM-HF study, NCT02032004), will hopefully help in establishing allogeneic MSC therapy as a standardized treatment modality in these patient cohorts.

## 10. Developing Approaches for Myocardial Regeneration in Patients with Ischemic and Non-Ischemic Heart Failure

Since the application of stem cell therapy has not yet yielded the desired results, the focus of the heart failure community is expanding to newer cell-based and non-cell-based methodologies of myocardial regeneration. Preclinical studies have been performed to evaluate the safety and preliminary efficacy of exosome therapy, genetic reprogramming, and the use of different biomaterials to potentiate the regenerative properties of stem cells [93].

Exosomes are small extracellular vesicles that are rich in cholesterol but also contain cytokines, proteins, and micro RNAs [94,95]. Several studies have suggested that exosome therapy may be as efficacious as standard stem cell therapy [96,97], as preclinical data suggest that exosomes may have a reparative potential in a diseased myocardium [97,98]. A cell-free approach to myocardial regeneration is also inviting because it circumvents several problems associated with the standard stem cell therapeutic approach, which include poor cell retention, immune response to allogeneic cells, arrhythmogenesis and oncogenesis. There are also preclinical data available to support the use of microRNAs in myocardial regeneration [99], as hsa-miR-590 and hsa-miR-199a were shown to promote the re-entry of “dormant” adult cardiomyocytes into the proliferation cycle [100,101]. In addition, miR302-367 expression was found to lead to an increase in cardiomyocyte mass, decreased myocardial fibrosis, and the improved function of a failing myocardium.

Building on previously elaborated cardiopoietic process [37], colleagues from the Mayo Clinic recently developed an approach to regenerate a damaged myocardium through genetic engineering [102]. They used the single gene modification technique to introduce an early cardiogenic gene into human adipose-derived mesenchymal stem cells. A single mesodermal transcription factor, Brachyury, was sufficient to trigger the high expression of cardiopoietic markers, Nkx2.5 and Mef2c. In a murine model of myocardial infarction, the intramyocardial delivery of engineered cardiopoietic stem cells (600,000 cells per heart) improved cardiac performance and protected against decompensated HF. Similarly, it has been shown by several research groups that fibroblast trans-differentiation into cardiomyocyte-like cells may yield a novel approach to myocardial regeneration [103]. Collectively, the data suggest that although the direct reprogramming of cells into beating cardiomyocytes is not feasible in vitro, trans-differentiation could produce new cardiomyocytes from endogenous cardiac fibroblasts and improve cardiac function after myocardial injury [104,105]. Somatic gene editing is a method that certainly opens up a cluster of new possibilities in the treatment of HF patients [106], yet technical challenges (viral vector immune response, nonviral vector delivery, and the size of the vector) and off-target safety concerns remain to be excluded, firstly in larger animals and then in humans.

The use of nanomaterials (in the form of nanostructured surfaces, nanoparticles, and nanocomposites) and biomaterials (such as tissue-engineered patches and heart matrix-derived injectable hydrogels) is another promising approach for myocardial regeneration that is predicating on ensuring the best microenvironment for the survival of stem cells and promoting their engraftment into the target myocardium. Nanomaterials have characteristics such as surface roughness, hydrophilicity, high surface energy, and reactivity that could lead to better protein adhesion and direct cell activities [107]. It was demonstrated that a 3D-printed patch composed of human cardiac-derived progenitor cells led to the long-term retention of more than 80% of implanted cells [108]. The use of 3D-printed bioscaffolds (made by inkjet printing, extrusion-based, and/or light-induced methods) [109,110] is new approach that could facilitate bioscaffold production and improve its performance while permitting the use of biofunctional complex tissue substitutes in each scaffold layer [111]. In addition, 2D materials can favorably mimic cardiac tissue, improve the viability of transplanted cells, allow for adequate electromechanical coupling, and deliver growth factors [107]. To date, constructing a bioscaffold thick enough and with a sufficient microvascular network to ensure the supply of the O_2_ and nutrients required for stem cell survival remains the biggest challenge with the use of biomaterials in myocardial regeneration and recovery [112]. Similarly, the use of an isolated cardiac extracellular matrix in 2D and 3D in vitro platforms has demonstrated the capability to provide tissue-specific signals for cardiac cell growth and differentiation [113]. The testing of the myocardial matrix hydrogel as a therapy after myocardial infarction in both small and large animal models has demonstrated improved left ventricular function, increased cardiac muscle mass, and cellular recruitment into the treated infarct [113]. Based on these results, hydrogels are currently being studied in the clinical setting to develop this technology for potentially wider clinical use in this patient cohort [114,115].

## 11. Lessons from the Past Studies to Be Considered in Designing Future Stem Cell Clinical Trials

Overall, there are several lessons that might be drawn from the current clinical experience with stem cell/regenerative therapy in HF patients.

1. A need for a multidisciplinary approach. Considering the ongoing paradigm shift, this would include a team consisting of an HF specialist, a cardiac imaging specialist, an interventional cardiologist, an expert in regenerative medicine, and experts in biomedical and genetic engineering. Such an approach would ensure that stem cells or other regenerative therapies are applied on top of the standard of care for HF patients, as well as that the applied therapeutic products are as optimal as possible.
2. The careful selection of patients for stem cell therapy. Currently, there are no widely accepted selection criteria for cell therapy in chronic HF patients, and the clinical predictors of response to cell therapy remain to be fully identified. As demonstrated by the CHART-1 trial, not all patients benefit from cell therapy to the same extent, as only patients with LV end-diastolic volumes between 200 and 370 mL benefited from cell therapy [39,116] These data emphasize the need for a patient-tailored approach, targeting those who have the highest likelihood of a beneficial response to cell therapy.
3. Standardized study endpoints need to be defined for phase III stem cell clinical trials. One of the important reasons for the divergent stem cell clinical data in chronic HF patients is the lack of a consensus on the endpoints that should be adopted in stem cell clinical trials. The transnational alliance for regenerative therapies in cardiovascular syndromes (TACTICS) international group summarized steps to consolidate preclinical research and recommended steps to design phase III/IV clinical trials [117]. Ideally, preclinical studies should be performed in large animals whose hearts resemble those of humans. The sample size should be large enough and endpoints need to be standardized through all phases of clinical trials in order to compare results from different studies. Hard endpoints are a necessity in phase III/IV clinical trials. Preferably, cardiovascular mortality should be emphasized, as typical populations in stem cell trials in the ischemic HF setting are older with various comorbidities that may increase all-cause mortality but mask the essential effect of cell therapy. Nevertheless, all-cause mortality should serve as safety readout and should be directionally concordant or at least neutral with the eventual improvement in cardiovascular mortality [118]. Surrogate endpoints are also important but have to reflect realistic improvements in a patient’s functional status and quality of life.
4. The therapeutic product intended for myocardial regeneration should ideally have the capabilities of inflammation modulation, fibrosis repair, angiogenesis stimulation, and de novo cardiomyocyte generation. Finding appropriate cellular or cell-free therapeutics that would have all of the abovementioned characteristics is a matter of ongoing and future research. However, given the cells’ biological complexity, in order to compare trials and their results, the standardization of biological products is needed. As stated in a recent document of the ESC working group on cardiovascular regenerative and reparative medicine, comparisons between different strategies or studies are only possible if standardized conditions regarding advanced biological product manufacturing, preclinical models, and target patient population profiles are used by different researchers [119]. This should be done according to a well-defined standardization and quality control strategy defined by authorities such as the European Medical Agency and American FDA.

## 12. Conclusions

In summary, different stem cell types, doses, and modes of cell delivery have been studied over the past two decades for the treatment of ischemic and non-ischemic HF patients. Although small-scale clinical trials have shown promising results with regard to the regional or global improvement in myocardial performance, myocardial scar reduction, and improvements in patients’ functional capacity and quality of life, these findings have not been uniform across trials and have not met initial expectations of both patients and physicians alike. Future stem cell therapeutic strategies should aim for a more standardized approach by establishing optimal stem cell type(s), dose, delivery method, and the stage of HF in which stem cell therapy would yield the best clinical response. In parallel with these standardizations, innovative regenerative therapy approaches should continue to be actively pursued and developed, as they will likely be needed to exploit the full potential of regenerative therapy in the HF patient cohort.

## Figures and Tables

**Table 1:** Randomized phase 2 and 3 trials in the ischemic HF setting.

Trial Name	No of pts	Cell Type(s)	Delivery Method	Primary Result
C-Cure [37]	45	Guided cardiopoietic BM-derived MSCs	Transendocardial	Significant improvement in LVEF and reduction in LV-end-systolic volume in the cell group (*p* < 0.0001)
Chart-1 [39]	271	Guided cardiopoietic BM-derived MSCs	Transendocardial	The primary efficacy endpoint was a Finkelstein–Schoenfeld hierarchical composite (all-cause mortality, worsening heart Failure, Minnesota Living with Heart Failure Questionnaire score, 6-minute walk distance, left ventricular end-systolic volume, and ejection fraction) that was neutral at 39 weeks (*p* = ns).
MSC-HF [34]	60	MSCs	Transendocardial	At 6 months, LVESV was reduced in the MSC group: −7.6 (95% CI −11.8 to −3.4) mL (*p* = 0.001) and increased in the placebo group: 5.4 (95% CI −0.4 to 11.2) mL (*p* = 0.07). There were also significant improvements in the LVEF of 6.2% (*p* < 0.0001), stroke volume of 18.4 mL (*p* < 0.0001), and myocardial mass of 5.7 g (*p* = 0.001) in the MSC group.
TRIDENT [45]	30	Allogeneic MSCs	Transendocardial	The ejection fraction only improved with 100 million by 3.7 U (*p* = 0.04). New York Heart Association class improved at 12 months in 35.7% (95% CI, 12.7% to 64.9%) with 20 million and 42.9% (95% CI, 17.7% to 71.1%) with 100 million. ProBNP (pro-brain natriuretic peptide) increased at 12 months with 20 million by 0.32 log pg/mL (*p* = 0.039) but not with 100 million (*p* = 0.65; between groups, *p* = 0.07).
Dib et al. [40]	23	Skeletal myoblast cells	Transendocardial	Improved LVEF and viability.
TAC-HFT [41]	65	MSCs and BMCs	Transendocardial	Over 1 year, the Minnesota Living With Heart Failure score improved with MSCs (*p* = 0.02) and BMCs (*p* = 0.005) but not with placebo (*p* = 0.38). The 6-minute walk distance only increased with MSCs. Infarct size as a percentage of LV mass was reduced by MSCs (*p* = 0.004) but not by BMCs (*p* = 0.11) or placebo (*p* = 0.36). Regional myocardial function as peak Eulerian circumferential strain at the site of injection improved with MSCs (*p* = 0.03) but not BMCs (*p* = 0.21) or placebo (*p =* 0.14). Left ventricular chamber volume and ejection fraction did not change.
Poseidon trial [36]	30	MSCs	Transendocardial	No changes in echo LVEF, improvement in 6MWT and MLWHFQ in treatment group.
Seismic trial [42]	47	Skeletal myoblasts	Transendocardial	At a 6-month follow-up, 6MW distance improved by 60.3 m in the treated group compared with no improvement in the control group (*p* = ns). In the control group, 28.6% experienced the worsening of heart failure status and 14.3% experienced an improvement in NYHA classification. Therapy did not improve global LVEF measured by MUGA at a 6-month follow-up.
Focus-HF [43]	30	BMCs	Transendocardial	Quality-of-life scores significantly improved at 6 months (*p* = 0.009 MLWHFQ and *p* = 0.002) over baseline in cell-treated but not control patients. Cell-treated younger patients had significantly improved maximal myocardial oxygen consumption (15 ± 5.8, 18.6 ± 2.7, and 17 ± 3.7 mL/kg per minute at baseline, 3 months, and 6 months, respectively) compared with similarly aged control patients (14.3 ± 2.5, 13.7 ± 3.7, and 14.6 ± 4.7 mL/kg per minute, respectively; *p* = 0.04)
Focus-CCTRN trial [44]	92	BMCs	Transendocardial	Changes in LVESV index (−0.9 mL/m(2) (*p* = 0.73)), maximal oxygen consumption (*p* = 0.17), and reversible defect (*p* = 0.84) were not statistically significant. There were no differences found in any of the secondary outcomes, including percent myocardial defect, total defect size, fixed defect size, regional wall motion, and clinical improvement.
HUC-Heart trial [35]	54	Human umbilical cord-derived MSCs and BMMNCs	Intramyocardial	LVEF only significantly increased (*p* = 0.004) in the HUC-MSC group (baseline to 12 months), as assessed with MRI. LVEF did not significantly change in the control (*p* = 0.139) or BM-MNC groups (*p* = 0.080). Cumulative analyses of LVEF change in patient-based analysis using MRI, SPECT, and Echo revealed no significant differences between the groups (*p* = 0.376, *p* = 0.110, and *p* = 0.765, respectively)
Science trial ongoing	138	Adipose-derived stem cell	Intramyocardial, NOGA guided	

MSCs—mesenchymal stem cells; BMC—bone marrow-derived stem cells; BMMNCs—bone marrow-derived mononuclear cells; LVEF—left ventricular ejection fraction; MLWHFQ—Minnesota Living with Heart Failure questionnaire; LVESV—left ventricular end-systolic volume; MRI—magnetic resonance imaging; SPECT—single-photon emission computed tomography.

**Table 2:** Clinical trials of stem cells in the DCMP setting.

Trial Name	No of pts	Cell Type(s)	Delivery Method	Study Endpoints
TOPCARE-DCM [70]	33	BMMC	Intracoronary	Change in regional LV wall motion of the target area
ABCD [71]	44 (24 cell-treated/20 controls)	BMMC	Intracoronary	Survival Change in NYHA functional class Change in LVEF Histopathologic evaluation
Bocchi et al. [72]	40 (23 cell-treated/17 controls)	BMMC	Intracoronary	Change in LVEF Change in exercise capacity Change in QoL
REGENERATE-DCM [73]	60 (15 peripheral G-CSF, 15 peripheral placebo, 15 IC stem cells, and 15 IC serum)	BMMC	Intracoronary	Change in global LVEF at 3 months Change in global LVEF at 12 months Change in exercise capacity Change in QoL Change in NT-proBNP
Vrtovec et al. [74]	55 (28 cell-treated/27 controls)	HSC (CD34^+^)	Intracoronary	Change in global LVEF at 12 months Change in exercise capacity Change in NT-proBNP
Vrtovec et al. [75]	110 (55 cell-treated/55 controls)	HSC (CD34^+^)	Intracoronary	Change in global LVEF at 12 months Change in exercise capacity Change in NT-proBNP
Vrtovec et al. [76]	40 (20 IC injections/20 TE injections)	HSC (CD34^+^)	Intracoronary/ Transendocardial	Change in global LVEF at 12 months Change in exercise capacity Change in NT-proBNP
Butler et al. [77]	22 (10 cell-treated/12 placebo)	alloMSC	Intravenous	Change in all-cause mortality Change in all-cause hospitalizations Adverse events Change in LVEF Change in exercise capacity Change in QoL Change in NT-proBNP
POSEIDON-DCM [78]	37 (19 alloMSC/18 autoMSC)	Allo vs. autoMSC	Transendocardial	Adverse events Change in LVEF Change in exercise capacity Change in QoL

## Data Availability

Not applicable.
